# Changes of adrenocorticotropic hormone rhythm and cortisol circadian rhythm in patients with depression complicated with anxiety and their effects on the psychological state of patients

**DOI:** 10.3389/fpsyt.2022.1030811

**Published:** 2023-01-18

**Authors:** Zheng Xie, Yajie Deng, Chunyu Xie, Yuanlong Yao

**Affiliations:** ^1^Department of Psychological Medicine, Henan Provincial People’s Hospital, People’s Hospital of Zhengzhou University, Zhengzhou, Henan, China; ^2^Henan Center for Disease Control and Prevention, Zhengzhou, Henan, China; ^3^Medical College of Henan University, Kaifeng, Henan, China

**Keywords:** artificial intelligence, depression, circadian rhythm, adrenocortical hormones, cortisol

## Abstract

**Objective**: This work was to explore the rhythm of adrenocorticotropic hormone (ACTH) and cortisol in patients with depression and anxiety and their effects on mental state. In this work, with depression complicated with anxiety patients as the A-MDD group (*n* = 21), and depression without anxiety symptoms as the NA-MDD group (*n* = 21). Firstly, data features were extracted according to the electroencephalo-graph (EEG) data of different patients, and a DR model was constructed for diagnosis. The Hamilton Depression Scale 24 (HAMD-24) was employed to evaluate the severity, and the ACTH and cortisol levels were detected and compared for patients in the A-MDD group and NA-MDD group. In addition, the psychological status of the patients was assessed using the Toronto Alexithymia Scale (TAS). As a result, the AI-based DR model showed a high recognition accuracy for depression. The HAMD-24 score in the A-MDD group (31.81 ± 5.39 points) was statistically higher than the score in the NA-MDD group (25.25 ± 5.02 points) (*P* < 0.05). No visible difference was found in ACTH levels of patients in different groups (*P* > 0.05). The incidence of cortisol rhythm disorder (CRD) in the A-MDD group was much higher (*P* < 0.05). The differences in TAS scores between the two groups were significantly statistically significant (*P* < 0.01). In conclusion, the AI-based DR Model achieves a more accurate identification of depression; depression with or without anxiety has different effects on the mental state of patients. CRD may be one of the biological markers of depression combined with anxiety.

## 1. Introduction

Depression is a common psychiatric disorder characterized by endless sadness and loss of interest in favorite things, usually accompanied by inability to perform normal daily activities for 14 days or longer (1). The incidence of depression has shown an upward trend year by year. In 2015, according to the estimates of the World Health Organization (WHO), there are currently more than 300 million people with depression in the world, and this number has been increasing in recent years (2). The new coronavirus that appeared in late 2019 swept the world, increasing the psychological pressure of some people, especially those in the epidemic areas, and the risk of depression. In addition to psychological problems, depression can also cause other physical diseases, causing patients to suffer from multiple problems. Depression is a major cause of illness or disability globally. In recent years, there have been continuous news of suicide, and some of them are patients with depression. If the patient is not relieved in the state of depression for a long time, it is likely to cause suicide and other behaviors (3).

With the improvement of people’s living standards, people pay more and more attention to depression. However, patients seeking a diagnosis of depression remain problematic. First, the diagnosis of depression still relies on the judgment of doctors, and the lack of a biomarker that can accurately identify depression makes the diagnosis subject to a certain degree (4, 5). Due to the lack of specific diagnostic methods for depression, its manifestations are diverse, and accurate and reliable diagnosis mainly depends on the clinical skills and clinical judgment of doctors. Comprehensive collection of accurate and reliable medical history data and extremely detailed physical and mental examinations are the basis for the diagnosis of depression (6). Psychiatrist typically conduct psychological assessments by asking about symptoms, thoughts, feelings, and behavioral patterns, and sometimes have patients fill out scales to assess and diagnose patients (7). At present, there is no recognized auxiliary test method for diagnosing depression. Due to its stability and complexity, there is currently no hospital in China that uses laboratory-developed diagnostic techniques in outpatient clinics. A doctor’s diagnosis is not absolutely objective. Secondly, it is inconvenient to seek a doctor’s diagnosis due to the embarrassment of speaking up or the ignorance of the dangers of depression. In fact, most depression patients do not receive timely and effective treatment at present (8).

Electroencephalo-graph (EEG) not only provides diagnostic basis for some brain diseases, but also provides accurate and effective treatment for some brain diseases. In order to diagnose depression more conveniently and simplify the process of EEG collection, an EEG recognition model with fewer channels is of great significance (9). In the context of the current hot research on artificial intelligence, machine learning methods have been widely applied, and the field of AI-assisted diagnosis and treatment has been developing rapidly (10). In addition, depression has received widespread attention in recent years. In this context, many researchers are trying to detect depression by machine learning using EEG signals at rest. The latest literature has proved that the use of resting state EEG detection and classifier is a feasible method to identify depression (11, 12). Depression also involves neurobiochemical, neuroendocrine, electrophysiological, neuroimaging and other changes. Although the pathogenesis of depression has been explored in various fields such as neuroendocrinology, imaging, and genomics, it is still not completely understood.

Current studies believe that depression is a disease with a certain biological basis, and its occurrence is the result of interaction of social psychological, physiological, and other factors (13). Chronic stress and high cortisol production also trigger a series of changes involving the serotonergic system, according to one study. These results suggest that changes in the endocrine system are related to the pathophysiology of anxiety and depression (14). In addition, the above results all prove that patients with depression and anxiety may have a dysfunctional HPA axis, but these results all give evidence of abnormal responses caused by exogenous stimuli. The HPA axis is the stress system in the body, including the hypothalamus, pituitary gland, and adrenal gland. In response to stress, neural stimulation is activated to secrete adrenocorticotropin-releasing hormone (CRH) from extracellular neurons in the paraventricular nucleus of the hypothalamus, thus stimulating the pituitary gland to release adrenocorticotropin-releasing hormone (ACTH) compared to depressive symptoms alone. Depression with anxiety has an obvious neurobiological feature, namely immune system and HPA axis dysfunction (15). Studies have shown that in patients with severe depression and anxiety, the expression of genes related to the expression of inflammatory factors is up-regulated (16). Among them, the key genes of leukocyte expression related to inflammation, cellular stress and emotional state are STMN1, p16ink4a, FOS, DUSP1, TERT, and IL-6 genes. Among these genes, FOS and DUSP1 respond better to stress and anxiety states (17).

Current research on the EEG recognition of depression has been carried out, but most of the research is collected from a small number of healthy people and depressed patients. This is related to the fact that currently the open source EEG samples are mainly healthy people, while the number of EEG samples of depressed patients is very small. In this context, in order to better understand the neurobiological characteristics of anxiety depression, further distinguish from depression without anxiety, to help the treatment of depression with anxiety patients. Therefore, this study will explore the rhythm of adrenocorticotropic hormone (ACTH) and cortisol in patients with depression and anxiety and their effects on mental state based on EEG characteristics, so as to provide clinical basis for the treatment of patients with depression and anxiety.

## 2. Materials and methods

### 2.1. Research objects

This work enrolled patients with depression who were hospitalized in the Henan Provincial People’s Hospital from June 2020 to June 2021.

According to the Hamilton Depression Scale (HAMD24), patients with anxiety/somatization factor score ≥ 7 were classified as depressed patients with anxiety and anxiety; and those with anxiety/somatization factor score ≤ 2 were classified as depressed patients without anxiety. Patients with depression and anxiety were selected as A-MDD group (*n* = 21), and patients with depression and no anxiety symptoms were selected as NA-MDD group (*n* = 21). This study was approved by the Ethics Committee of the Hospital. The purpose, methods, and requirements of this study were explained to patients with depression who met the inclusion criteria and their families. After obtaining the consent of patients and their guardians, the patients signed informed consent to participate in this experimental study. The inclusion and exclusion criteria were shown in Tables 1, 2.

**TABLE 1:** Inclusion criteria for patients with depression.

No.	Inclusion criteria
1	Meeting the DSM-5 diagnostic criteria for MDD
2	HAMD24 score ≥ 21 points
3	No history of using immunosuppressants in the past 6 months, and no history of substance dependence or abuse
4	No history of serious medical illness, no history of infectious disease within the past 2 weeks
5	Han nationality, aged 18–65 years old, primary school or above
6	No antidepressant use in the past week
7	Informed consent of subjects or their guardians

**TABLE 2:** Exclusion criteria for patients with depression.

No.	Exclusion criteria
1	Persons of non-Han nationality among themselves or blood relatives
2	Mental retardation and dementia
3	Complicated with definite heart, lung, brain, kidney, and other important organ diseases
4	Women who were pregnant or breastfeeding
5	Patients requiring glucocorticoid therapy

### 2.2. Scales and tables

#### 2.2.1. Data collection form

The diagnosis was determined by cross-consultation between two or more senior psychiatrists (associate chief physicians and above). A self-compiled collection table of general data and clinical features was used. General information and clinical characteristics collected included name, sex, age, contact information, years of education, age of onset, course of disease, body mass index (BMI), systolic blood pressure (SBP), and diastolic blood pressure (DBP). Circadian rhythm disturbances were defined as 50% ACTH or COR 16 ≥ 8, or 50% ACTH or COR 24 ≥ 16, or 24 ≥ 8. Therefore, venous blood was taken by venous puncture at 8:00, 16:00, and 24:00 every day, and plasma levels of ACTH and COR were measured.

#### 2.2.2. HAMD scale

Developed by Hamilton, the scale is one of the commonly used clinical tools for assessing the severity of depression. At present, the scale is developed by three versions: 17, 21, and 24 items. Among them, the 24-item (HAMD-24) is the most comprehensive, so it was adopted in this work. The scale had good reliability and validity. It was suitable for evaluating adult patients with depressive symptoms or depression. It was commonly used to assess depressive symptoms in bipolar disorder, depression, and other disorders. The severity of the disease was judged by the total score. The milder the symptoms, the lower the total score. Conversely, the more severe the symptoms, the higher the overall score. A HAMD-24 score of more than 35 points indicated severe depression; a total score of 20–35 points: definitely depression; a score of 8–20 points: possible depression; and a score of less than 8 points: normal.

#### 2.2.3. Toronto Alexithymia Scale (TAS)

Toronto Alexithymia Scale (TAS) was compiled by Taylor et al. and was introduced into China in 2003. The translation of TAS-20 and the reliability and validity of the Chinese version of TAS were carried out. It was found that the test-retest correlation coefficient of each subscale was 0.687–0.893, indicating that the scale had good stability across time. The internal consistency coefficients ranged from 0.581 to 0.739, and the split-half correlation coefficients of the subscales ranged from 0.558 to 0.803. In this work, the Chinese version of TAS-20 was adopted, which consisted of 20 items with three factors: difficulty in identifying feelings (DIF), difficulty in describing feelings (DDF), and externally oriented thinking (EOT). Extroverted thinking referred to the lack of the ability to reveal inner attitudes, feelings, and desires, and clinging to the minutiae of external things. It was difficulty to identify self-emotion factor includes seven items 1, 3, 6, 7, 9, 13, and 14; difficulty to describe self-emotion factor includes five items 2, 4, 11, 12, and 17; and the extraverted thinking factor included 5, 8, 10, 15, 16, 18, 19, and 20 items. The Chinese version of TAS-20 was a self-rating scale with a five-point scale (1 = strongly disagree, 2 = disagree, 3 = partially agree, 4 = agree, and 5 = strongly agree). Among them, 4, 5, 10, 18, and 19 were reverse scoring. The total score of the scale was between 20 and 100, and the higher the total score, the more serious the alexithymia. A total score of ≤ 51 indicated non-alexithymia; a total score of 52 ≤ 51 indicated moderate alexithymia; and a total score of ≥ 61 indicated severe alexithymia.

#### 2.2.4. Scale consistency evaluation

All researchers were psychiatrists, and the researchers must master the inclusion and exclusion criteria of the two groups of patients, the process of the subject, and the scale evaluation method (18, 19). The evaluation method adopted a combination of inquiry and observation, and the enrolled patients were scored strictly according to the content of the scale. Individual items needed to be collected from family members and medical staff. Each assessment was about 15–20 min, and the patient’s condition was assessed at the time or 1 week before enrollment. The consistency check kappa value was 0.85.

### 2.3. Depression-related EEG features

To find EEG features related to depression, this work referred to a large number of literatures, and finally determined the features that need to be calculated. In this work, a variety of feature calculations are involved, including linear features, non-linear features, and power spectrum features, which were possible markers for depression identification.

#### 2.3.1. Linear features

The first step was to find the maximum value in this EEG sequence (20).

Then it should calculate the variance in each piece of EEG data. Variance can measure the dispersion of EEG sequence data (21).

Kurtosis is also known as the kurtosis coefficient. The number of features representing the peak height of the probability density distribution curve at the mean value (22). In statistics, kurtosis was used to measure the probability distribution of real random variables. The Kurtosis of a set of sequences, the specific calculation method was shown in Equation (1).


(1) $$\text{Kurtosis}=\frac{1}{n-1}\,\sum_{i=1}^{n}\frac{{\left(x_{i}-\bar{x}\right)}^{4}}{S\,D^{4}}-3$$

In the above equation, $\bar{x}$ represents the sample mean and SD represents the standard deviation, i.e., $\sqrt{V\,a\,r\,i\,a\,n\,c\,e}$. In practical applications, the kurtosis value is usually subtracted by 3, so that the kurtosis of a normally distributed series is 0. Kurtosis was equal to 0, indicating that the overall distribution of the series is the same as the normal distribution. If the standard deviation and mean of

was larger, which meant that this series has more maxima and minima, that is, extreme values.

Finally, skewness was a feature used to measure the direction and degree of skewness in the distribution of statistical data. The definition of skewness was as follows, where k2 and k3 represented the second-order and third-order central moments, respectively, and σ represents the standard deviation, as shown in Equation (2).


(2) $$\mathrm{Skew}\,\left(X\right)=E\,\left({\left(\frac{X-\mu }{\sigma }\right)}^{3}\right)=\frac{{\text{k}}^{3}}{\sigma^{3}}=\frac{{\text{k}}_{3}}{{\text{k}}_{2}^{3/2}}$$

If the skewness was expressed by bs, and the bs of the data was less than 0, the distribution of the data was said to have a negative deviation, and the data on the left of the mean was less than the data on the right. A bs greater than 0 was the exact opposite of a negative deviation. When the skewness was equal to 0, the distribution of the sample can be considered to be symmetric. Skewness can be used to detect normality of distributions.

#### 2.3.2. Non-linear features

Detrended volatility analysis (DFA) method was used to analyze the long-range correlation of time series. One of the advantages of the DFA method was that it can filter out the trend component of each order in the signal sequence and can detect long-range correlation and polynomial trend signals superimposed with noise. It was suitable for long-range power-law correlation analysis of non-stationary time series.

Computing the DFA started by integrating the sequence data. Let the original sequence be X-[X(1), X(2),…,X(N)]. The definition of the integral signal Y = [Y(1), Y(2),…, Y(N)] was shown in Equation (3) below.


(3) $$Y\,\left(k\right)=\sum_{i=1}^{i=k}\left(x\,\left(i\right)-\mathrm{Ave}\right)$$

In the equation above, Ave represented the mean of the sequence x. The second step was to slice the integral sequence Y into boxes. A minimum of two boxes were required to compute the DFA. In practical applications, it is usually from 1/5 of the signal length to (x-5) of the signal length, where x was the nearest power of 2 of the signal length, i.e., 1/16, 1/32, 1/64, 1 /128,…. In each box, a linear least squares fit was applied to the data in the box. Denote the series on the assembly line as its kth element *Y*_*n*_(k) corresponding to y(k). For the fit in each box, there was a remainder, the sum of squares of all offsets, and the difference between the actual point and the point on the fitted line. F(*n*) represented the square root of the average total remaining in all boxes when the box length was *n*, so there were Equations (4) and (5).


(4) $${\mathrm{Total}}_{R}\,\mathrm{esidue}=\sum_{k=1}^{k=n}\left(\text{y}\,\left(\text{k}\right)-Y_{n}\,\left(\text{k}\right)\right)$$

(5) $$F\,\left(n\right)=\sqrt{\left(\mathrm{Total}\,\_\,\mathrm{Residue}/n\right)}$$

The calculation of F(*n*) was performed for each box length n, resulting in the relationship between *n* and F(*n*). In general, F(*n*) increased as n increased. Finally, the relationship between F(*n*) and *n* was analyzed. A least squares fit was performed between log[F(*n*)] and log(*n*). The slope of the fitted line was the DFA value, denoted as alpha. For white noise, the value of alpha should be 0.5. If a signal had a higher complexity, then its alpha value was also higher.

Higuchi Fractal Dimension (HFD) was related to entropy, and entropy was directly related to the amount of information in the signal. Fractal dimension can be simply interpreted as the degree of curvature or irregularity of a piece of signal. In addition, the HFD was undertaken as an important feature extraction method. For a limited set of time series data S(1), S(2), S(3)…S(*n*), a new time series can be obtained according to the following equation, as shown in Equation (6).


(6) $$S_{k}^{m};S\left(m\right),S\left(m+k\right),S\left(m+2k\right),\ldots \ldots S\left(m+\left[\frac{N-1}{k}\right].k\right)$$

m and k were integers, representing the start time and interval time, respectively. Furthermore, the curve length was defined as Equation (7).


(7) $$L_{m}\,\left(k\right)=\frac{1}{k}\,\sum_{i=1}^{\frac{N-m}{k}}\left|X\,\left(m+\mathrm{ik}\right)-X\,\left(m+\left(i-1\right)\right)\cdot k\right|\,\frac{N-1}{\left(\frac{N-m}{k}\right)\cdot k}/k$$

Mean of *L*_*m*_(*k*) was calculated with Equation (8).


(8) $$L\,\left(k\right)=\sum_{m}^{k}L_{m}\,\left(k\right)$$

Spectral Entropy was to describe the relationship between power spectrum and entropy rate. Its calculation process was as follows:

Let Y be the input of a stable system, and the system transfer function was shown in Equation (9).


(9) $$G\,\left(e^{-\mathrm{i2}\,\pi \,f}\right)=G\,{\left(B\right)}_{B=\exp\left(-\mathrm{i2}\,\pi \,f\right)}$$

x_*t*_ was the stationary output of the system, then the relationship between the input and output entropy rate of the system could be given as follows:


(10) $$h_{x}=h_{y}+\frac{1}{2}\int_{\frac{1}{2}}^{\frac{1}{2}}In\;|G(f){|}^{2}df$$

In time series, this relationship was most commonly seen with input {y_*t*_} zero-valued white noise. The variance was $\sigma_{y}^{2}$, then the transfer function of the system was ${\left|G\,\left(f\right)\right|}^{2}=\frac{G_{x}\,\left(f\right)}{\sigma_{y}^{2}}$, and the entropy rate of white noise was expressed in Equation (11).


(11) $$h_{y}=\ln\sigma \,y\,\sqrt{2\,\pi \,e}$$

Therefore, the entropy rate of {x_*t*_} can be calculated as Equation (12).


(12) $$h_{y}=In\;\sqrt{2\pi e}+\frac{1}{2}\int_{\frac{1}{2}}^{\frac{1}{2}}In\;G(f)df$$

Equation (12) reflected the relationship between the spectral density of {x_*t*_} and the encapsulation ratio. Among them, $\ln\sqrt{2\,\pi \,e}$ was a constant, and the larger *h*_*x*_ led to larger $\frac{1}{2}\,\int_{\frac{1}{2}}^{\frac{1}{2}}\ln G\,\left(f\right)\,d\,f$. Therefore, the sub $\int_{\frac{1}{2}}^{\frac{1}{2}}\ln G\,\left(f\right)\,d\,f$ was called the spectral entropy of the signal {x_*t*_}, that is, the power spectrum was to represent the entropy. Spectral entropy was related to the power spectrum, but was still a kind of entropy, which was classified as a non-linear feature here.

### 2.4. EEG data collection

**FIGURE 1:**
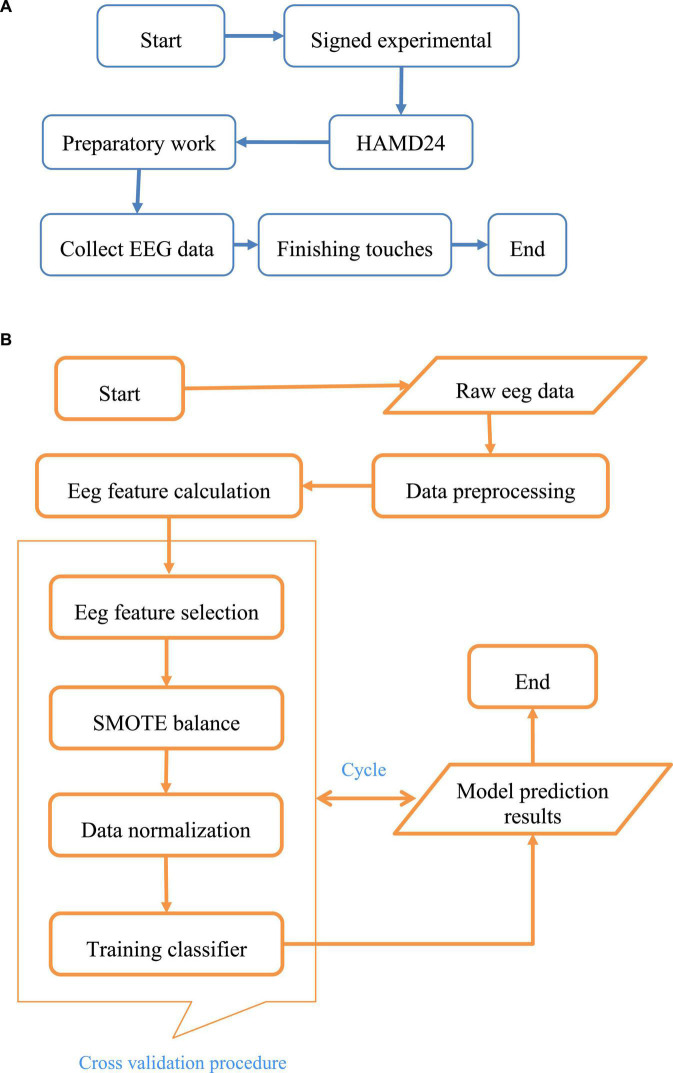
The process of EEG data collection and identification for depression. Among them, **(A)** was the flow chart of EEG acquisition, and **(B)** showed the experimental research flow of the depression recognition model.

Although there were studies on EEG identification of depression, most of the studies involved a small number of EEG samples, even only 10–20 EEG data of patients with depression, and none of them were open-source. The open-source EEG datasets on the Internet were mainly based on the data of healthy people, and the EEG data of patients with depression were very scarce, so the datasets used in this work needed to be collected by themselves (23, 24). In The patient’s quiet state, the collection process was shown in Figure 1.

Among them, A was the flow chart of EEG acquisition, and B showed the experimental research flow of the depression recognition model.

### 2.5. Design of DR model

**FIGURE 2:**
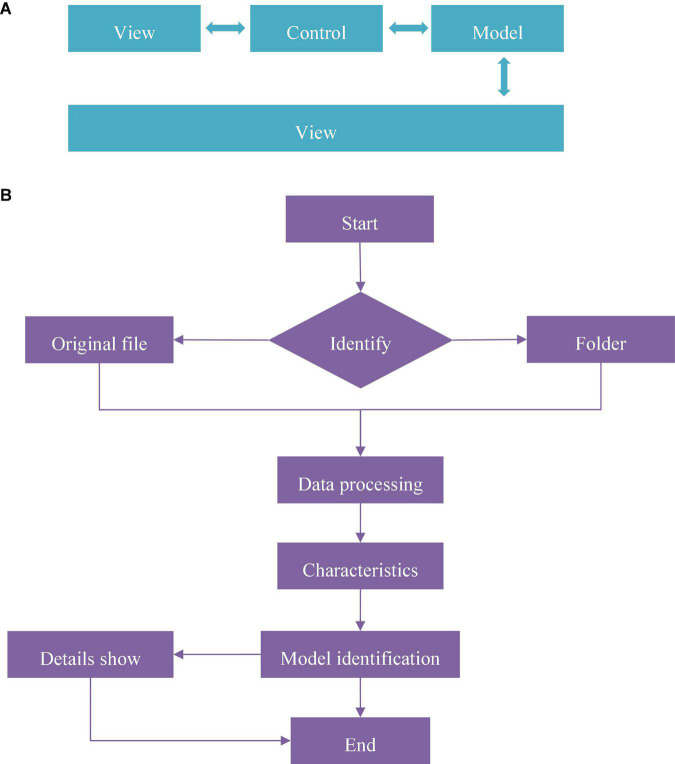
The design process of DR model. **(A)** Was the overall structure of the system and **(B)** showed the main program flow chart.

The overall structure of the system can be divided into four layers. The top layer was the user interaction layer (View), which mainly included user input files and the output of depression recognition results. The business layer (Control) was followed, which was responsible for reading the user’s EEG files, calculating features, and identifying depression. The next layer was data layer (Model), which mainly included the calculation of some EEG intermediate results and so on. For persistent use, some Model data not only existed in memory but also be included in the disk synchronously. The bottom layer was the host environment and base libraries. In the overall design, the code structure of the DR model conformed to the MVC framework to achieve the effect of separating the business model from the user interface. The system recognition included the process from the user starting the program and inputting the EEG signal until the recognition result appeared. The system also included an optional option, whether to view the details of your EEG analysis. The process logic used this option to determine whether to display the details. The specific design process of DR model was given in Figure 2.

### 2.6. Statistical analysis methods

Quantitative data were obtained by *t*-test with two independent samples: cortisol, adrenocorticotropin, C-reactive protein (CRP), blood lipid, etc. The difference between A-MDD and NA-MDD was compared. Chi-square test was used to test gender and family history, and the differences between the two groups were analyzed. Multivariate Logistic regression was used to analyze the effects of age, BMI, blood pressure, CRP, blood lipid, ACTH level, and 7 HAMD scores on depression and anxiety. SPSS 20.0 software was used to analyze the data. The graph shows the mean and standard deviation.

## 3. Results

### 3.1. Recognition results of the model

**FIGURE 3:**
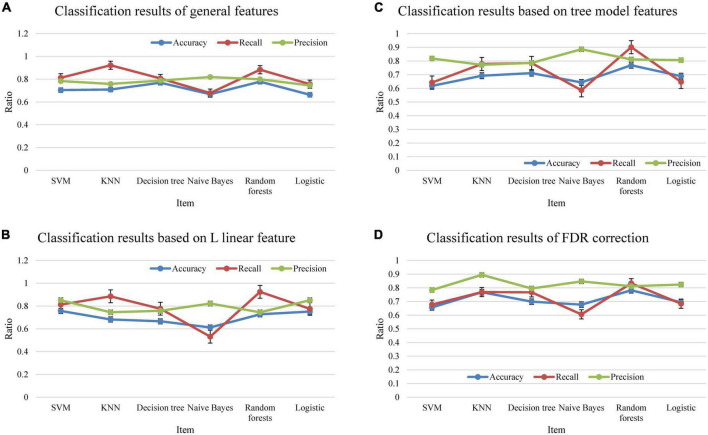
The classification results. **(A)** Showed all feature results, **(B)** showed the linear feature result based on L, **(C)** was the feature result based on tree model, and **(D)** showed the FDR correction result.

The evaluation indicators that this work focused on were Accuracy, Recall, and Precision. The results of 10 consecutive fivefold cross-validation were illustrated in Figure 3.

### 3.2. Comparison on general data

**FIGURE 4:**
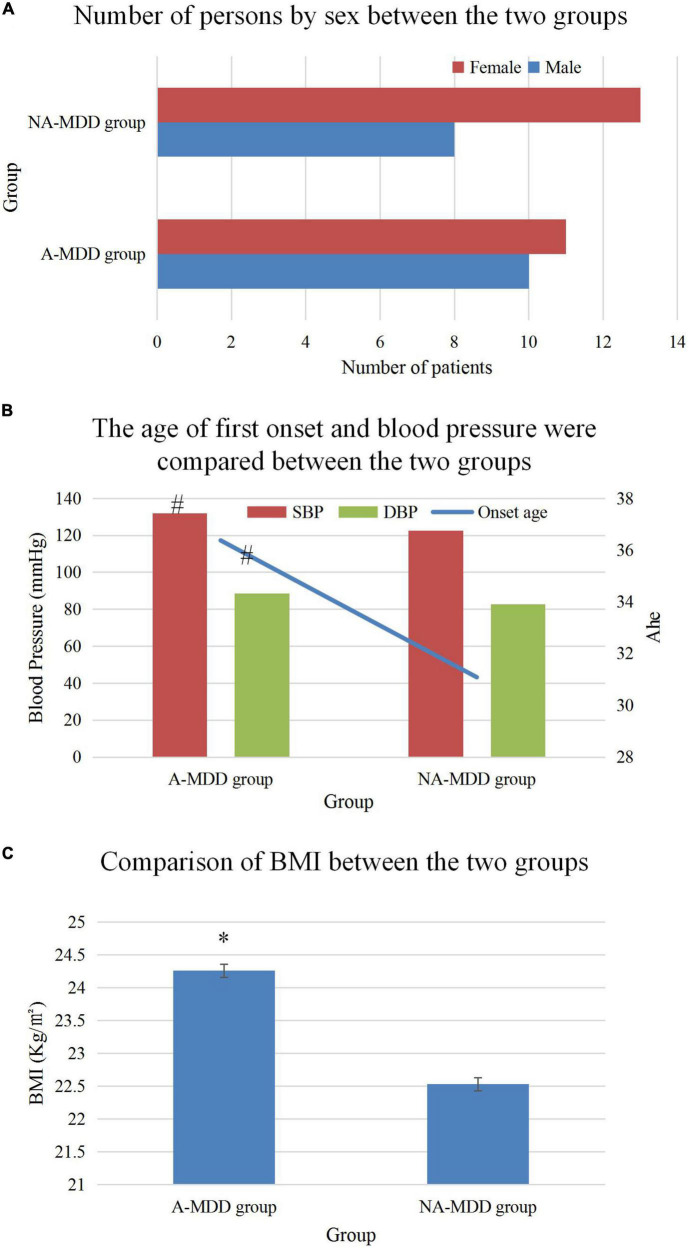
Comparison of general data of patients. **(A)** Was the comparison of the number of men and women, **(B)** showed the comparison of the first onset age and blood pressure, and **(C)** illustrated the comparison of the BMI value. * and ^#^Represented *P* < 0.05 and *P* < 0.01 compared with the NA-MDD group, respectively.

Comparison on the general data of the two groups of patients revealed that the first age, BMI and SBP in the A-MDD group were higher than those in the NA-MDD group (*P* < 0.05). There was no great difference in gender, course of disease, DBP, and family history of mental illness (*P* > 0.05). The details were given in Figure 4.

### 3.3. Comparison of HAMD-24 total score and factor scores

**FIGURE 5:**
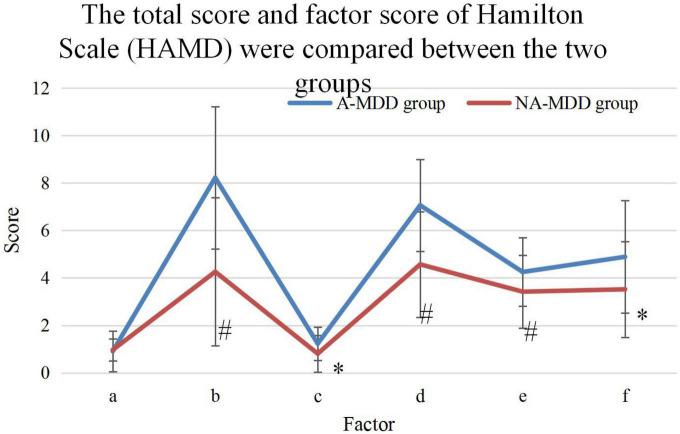
Comparison of the total and factor scores of the HAMD. * and ^#^Represented *P* < 0.05 and *P* < 0.01 compared with the NA-MDD group, respectively.

The total score and factor scores of HAMD were compared, and the results (Figure 5) found that the A-MDD group HAMD total score, cognitive impairment factor (a), day and night change factor (b), retardation factor (c), sleep obstacle factor (d), and hopelessness factor (e) were higher than those in NA-MDD group (*P* < 0.05). No statistically visible difference was found in body weight factor (f) (*P* > 0.05).

### 3.4. Comparison of ACTH and COR release levels and incidence of CRD

**FIGURE 6:**
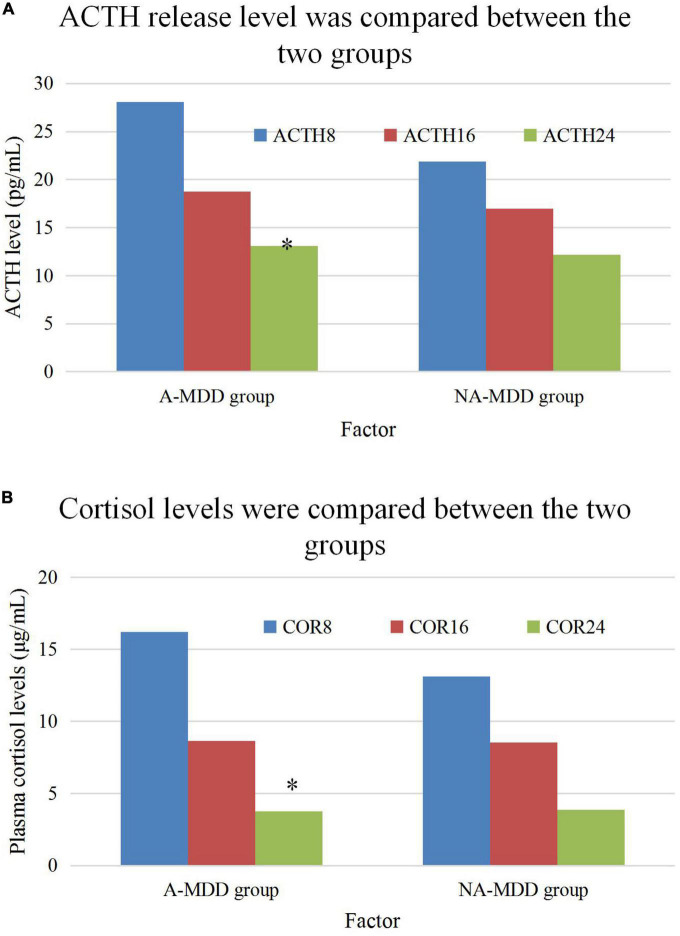
Comparison of ACTH and cortisol plasma levels. **(A)** Is the comparison of ACTH level and **(B)** is the comparison of cortisol plasma level. *Meant *P* < 0.05 compared with the NA-MDD group.

Figure 6 compared the plasma ACTH and COR levels at 8:00, 16:00, and 24:00 of patients in the A-MDD and NA-MDD groups. It was found that COR8 in the A-MDD group was higher (*t* = 2.003, *P* < 0.05); and no observable difference in plasma ACTH and COR levels was found at other time points (*P* > 0.05). The diurnal cortisol slope referred to the rate of decline of plasma cortisol concentration from morning to night. The A-MDD group showed obviously higher diurnal cortisol slope than the NA-MDD group (*t* = 2.06, *P* < 0.05).

**FIGURE 7:**
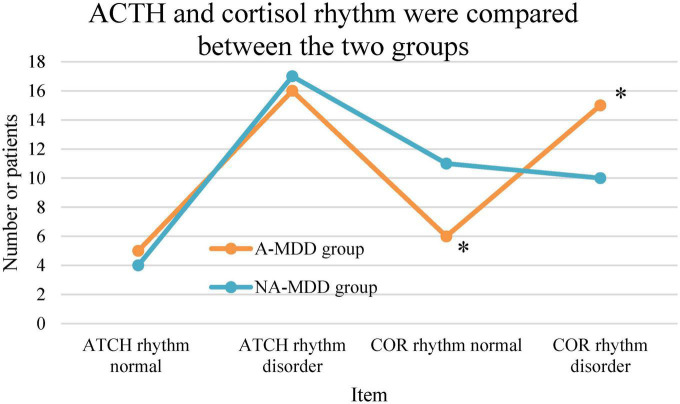
Comparison of ACTH and cortisol rhythms. *Meant *P* < 0.05 in contrast to the NA-MDD group.

Statistical results showed that A-MDD group the incidence of CRD of 72%, and the NA-MDD group showed the incidence of 51%; and the incidence of CRD was higher in the A-MDD group (x^2^ = 5.369, *P* = 0.02). In addition, there was observable difference between the two groups in the incidence of abnormal ACTH rhythm (*P* > 0.05), as shown in Figure 7.

### 3.5. Comparison of CRP levels between two groups

**FIGURE 8:**
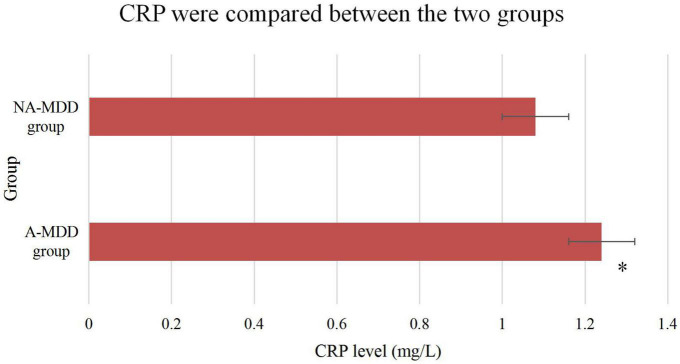
Comparison of CRP between the two groups. *Meant that compared with the NA-MDD group, the difference was statistically significant (*P* < 0.05).

The plasma CRP levels between the two groups were compared. It was found that the plasma levels of the A-MDD group were significantly higher than those of the NA-MDD group, and the differences were statistically significant (*P* < 0.05), as shown in Figure 8.

### 3.6. Comparison of TAS scores between the two groups

**FIGURE 9:**
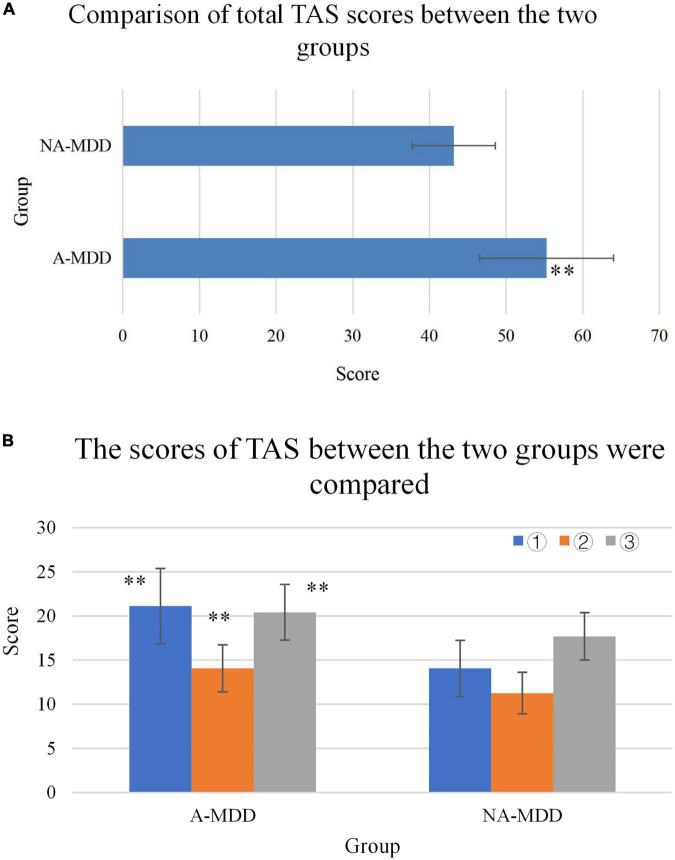
**(A)** Comparison of TAS scores between the two groups. **(B)** ^**^Represented a very significant difference compared with the NA-MDD group (*P* < 0.01).

The proportion of alexithymia individuals in the A-MDD group was 66.67%, while that in the NA-MDD group was 3.23%, and the difference between the two groups was statistically significant (*P* < 0.01). The total TAS score of the A-MDD group was significantly higher than that of the NA-MDD group, and the difference between the two groups was extremely significant (*P* < 0.01). The A-MDD group’s difficulty in identifying self-emotional factors was significantly higher than the NA-MDD group, and the difference between the two groups was extremely significant (*P* < 0.01). The A-MDD group was significantly higher than the NA-MDD group in terms of difficult to describe self-emotional factors, and the difference between the two groups was extremely significant (*P* < 0.01). The A-MDD group’s extroverted thinking factor score was significantly higher than that of the NA-MDD group, and the difference between the two groups was extremely significant (*P* < 0.01). The details were shown in Figure 9 (difficulty in identifying one’s own emotions, difficulty in describing one’s own emotions, and extraverted thinking corresponded to ➀, ➁, and ➂, respectively).

## 4. Discussion

In today’s society, more and more people are suffering from depression. The outbreak that began in late 2019 has also exacerbated depression. Depression has received more and more attention in today’s social life. Long-term depression will affect the patient’s study and work, their own body immunity will decline, induce colds, coronary heart disease and even tumors, and have a significant impact on both physical and mental aspects, including social functions. The terrible impact of depression also has a small number of patients with suicidal thoughts and thoughts, and some patients will eventually succeed in suicide, so they suffer from depression and must receive timely and effective treatment (25–27). The DR model based on EEG provided more objective help and reference for the diagnosis of depression, and had very important research value. In recent decades, with the vigorous development of machine learning, the rise of artificial intelligence-assisted diagnosis and treatment has provided a solid foundation for EEG identification of depression (28). At present, studies on EEG identification of depression have been carried out, but most of the studies are based on the collected EEG signals of a small number of healthy people and patients with depression. This is related to the fact that the current open-source EEG samples are mainly from healthy people, while the number of EEG samples from patients with depression is very small (29). This work not only involved a relatively large number of samples, but also found fewer EEG channels to identify depression, making the study a lot of work and challenging.

In this study, a depression recognition system based on EEG was constructed, and the results of previous experiments were integrated into the system. The system is implemented by multithreading and it is time-consuming to suppress the analysis calculation and intermediate result access. Separating the user interface thread from the feature computation thread effectively avoids user interface blocking. Using the system, users can input various types of EEG files, wait a few minutes, and get the results of depression identification and the probability given by the model. This result can be used as one of the indicators of depression and assist the clinical diagnosis of depression. A total of 5,518 possible depression related characteristics were calculated. After feature selection, the method of SMOTE balance sample + feature normalization + machine learning classifier was selected. Through repeated experiments and adjustment, it was found that an EEG classification model involving fewer EEG channels was found through the verification results of the A-MDD group. The experimental results show that the tree-based feature selection method can achieve classification accuracy of nearly 80% (79.61%) when only 12 features are used (involving 10 EEG channels).

This work found that depression with anxiety and simple depression had different clinical characteristics: HAMD total score, cognitive impairment, sleep disturbance, and hopelessness were more severe than those without anxiety. Some studies have also suggested that the treatment effect of anxiety-type depression is poor, and the drug side effects are more, suggesting that anxiety-type depression may be a subtype of depression with unique biological characteristics. In addition, this work found that patients with depression and anxiety had a later age of onset and a longer course of disease, suggesting that patients with this subtype may be more affected by social and psychological factors in the pathogenesis, which is consistent with the research of some domestic scholars (30). Depression complicated with anxiety, a specific subtype of depression, is associated with poorer clinical outcomes, such as worse psychosocial functioning and treatment response. Therefore, uncovering the neurobiology of this subtype is particularly important for improving diagnosis, prognosis, and treatment (31). This work also found that BMI, SBP, and triglyceride in patients with depression complicated with anxiety were significantly higher than those in patients with depression. Cortisol rhythm disorder (CRD) may be a biological marker of depression complicated with anxiety. There are obvious circadian changes in the HPA axis in the normal population, and the circadian system is not only considered an important regulator of systems that play a role in the development of mood disorders.

HPA axis dysfunction is one of the most common neuroendocrine abnormalities in patients with depression. About 60% of patients with depression have increased glucocorticoid secretion, that is, HPA axis hyperfunction, strong pituitary secretion, and significantly increased plasma cortisol. High levels of plasma cortisol can promote the occurrence and development of depression to a certain extent, leading to aggravation of depression. It was found in this work that the TAS scores of the two groups of patients had extremely significant differences (*P* < 0.01). Relevant studies have shown that adaptive emotion regulation is significantly negatively correlated with depression level in patients with depression, but not with anxiety level (32). This may indicate that patients with depression who tend to use adaptive emotion regulation strategies have relatively low levels of depression, and the individual’s adaptive emotion regulation is a protective factor. Positive refocusing and positive reappraisal were also significantly negatively associated with depression levels in patients with depression. That is, the ability to shift perspective, to step away from negative emotional events and focus on other, less negative events, may be associated with relatively low levels of depression in depressed patients (33). Also, patients who are able to change their perspective, see the positive meaning of things, or change their perception of the impact of events, may experience lower levels of depression.

## 5. Conclusion

This work found that the AI-based DR model had a higher recognition accuracy for depression; the HAMD score of patients in the A-MDD group was notably higher than the score of patients in the NA-MDD group. The incidence of cortisol circadian disturbance was much higher in the A-MDD group; there was a difference in TAS scores between the two groups. Therefore, it was believed that the AI-based DR Model achieves more accurate results in identifying depression; depression with or without anxiety symptoms had different effects on the mental state of patients; and the incidence of CRD in patients with depression and anxiety was higher than that in patients with depression alone. CRD can be used as one of the biological markers of depression and anxiety.

## Data availability statement

The original contributions presented in this study are included in the article/supplementary material, further inquiries can be directed to the corresponding author.

## Ethics statement

This study was approved by the Ethics Committee of Henan Provincial People’s Hospital. The patients/participants provided their written informed consent to participate in this study.

## Author contributions

ZX, YD, CX, and YY participated in the conception, designed the study, and involved in the data collection and analysis of the study. CX prepared the manuscript for publication. ZX, YD, and YY critically reviewed the manuscript. All authors read and approved the final manuscript.
